# Individualized brain-computer interface for people with disabilities: a review

**DOI:** 10.3389/fnhum.2026.1738876

**Published:** 2026-02-10

**Authors:** Simanto Saha, Petra Karlsson, Collin Anderson, Omid Kavehei, Alistair McEwan

**Affiliations:** 1 School of Biomedical Engineering, The University of Sydney, Sydney, NSW, Australia; 2 Cerebral Palsy Alliance, Discipline of Child and Adolescent Health, The University of Sydney, Sydney, NSW, Australia; 3 School of Medical Sciences, The University of Sydney, Sydney, NSW, Australia; 4 Cerebral Palsy Alliance, The University of Sydney, Sydney, NSW, Australia

**Keywords:** assistive and rehabilitative technologies, brain-computer interface, generalized algorithms, neuroimaging techniques, user-centric design

## Abstract

Brain-computer interfaces (BCIs) facilitate functional interaction between the brain and external devices, enabling users to bypass their typical peripheral motor actions to control assistive and rehabilitative technologies (ARTs). This review critically evaluates the state-of-the-art BCI-based ARTs by integrating the psychosocial and health-related factors impacting user needs, highlighting the influence of brain changes during development and aging on the design and ethical use of BCI technologies. As direct human-computer interfaces, BCI-based ARTs offer extended degrees of freedom via augmented mobility, cognition and communication, especially to people with disabilities. However, the innovation in BCI-based ARTs is guided by the complexity of disability types and levels of function across users that define individual needs. Therefore, an adaptable design is essential for tailoring a BCI-based ART that can fulfill user-specific requirements, which may hinder the scalability of BCIs for their widespread adoption across users with disabilities. The trade-offs between implantable and non-implantable BCIs are explored along with complex decisions around informed consent for people with communication or cognitive disabilities and pediatric settings. Non-implantable BCIs offer broader accessibility and transferability across users due to wider standardized signal acquisition and algorithm generalization, making them suited for a more comprehensive user group. This review contributes to the field by providing individualized user needs-informed discussion of BCI-based ARTs, emphasizing the need for adaptable designs that align the evolving functional and developmental needs of users with disabilities.

## 1 Introduction

**Figure 1:**
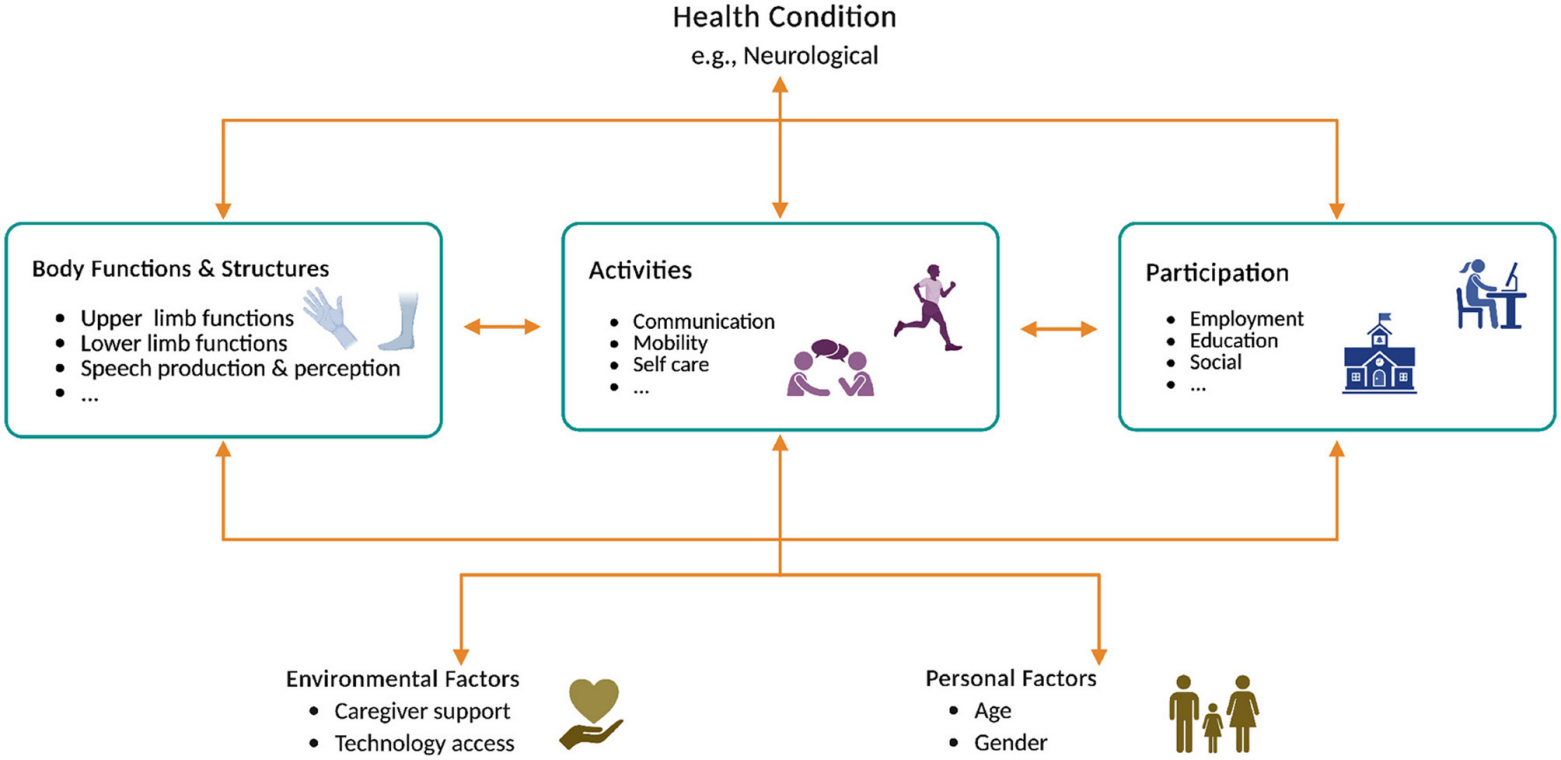
A schematic illustration of the World Health Organization's International Classification of Functioning, Disability and Health (ICF) framework to explain psychosocial factors impacting people with neurological conditions. Created in BioRender (https://BioRender.com/i4wp1su) licensed under CC BY 4.0.

Neurological disorders such as stroke, spinal cord injury, amyotrophic lateral sclerosis, attention deficit hyperactivity disorder, Alzheimer's disease, Parkinson's disease, and cerebral palsy can lead to functional disabilities and often involve associated impairments that affect communication, cognition and mobility, ultimately impacting quality of life (Metzger et al., 2023; Bekteshi et al., 2023; Hallett et al., 2022; Monforte et al., 2021; Makris et al., 2021; Micera et al., 2020; Goulet et al., 2019; Milekovic et al., 2018; Kasahara et al., 2018; Goldstein and Abrahams, 2013; Lim et al., 2012; Liberati et al., 2012). Multiple factors, from personal and environmental contexts to social and welfare elements, impact people with disabilities and their ability to participate in everyday life. The International Classification of Functioning, Disability and Health (ICF), developed by the World Health Organization (2002, 2007) (WHO), provides a comprehensive biopsychosocial model for understanding disability. It categorizes functioning across three domains: body functions and structures, activities and participation, and environmental and personal factors. This framework applies universally across all health conditions and emphasizes the interaction between health conditions and contextual factors in shaping individual functioning. Figure 1 schematically illustrates the complex interplay between the diverse psychosocial and health-related factors for assessing diverse health conditions, such as neurological conditions causing disabilities (Andresen et al., 2016; Hill et al., 2015; Mohammadi et al., 2023; Zhu et al., 2022; Edughele et al., 2022; Stamford et al., 2015). Neurological conditions can affect individuals in anatomical, physiological, psychological and cognitive domains, causing different impairments. The type and severity of impairments characterize the residual functional abilities of people with disabilities to participate in daily life activities. Diversity in impairments and disabilities combined with environmental and personal factors shapes highly individualized user needs, promoting the necessity of user-centric design of brain-computer interface (BCI)-based assisitive and rehabilitative technologies (ARTs) that fulfils user needs. Considering the psychosocial and health-related factors that influence individual needs is critical. Studies investigated users' basic characteristics such as gender and age, and aspects of operating BCI in naturalistic environments such as living situation, caregiver support and insurance coverage and their influence on BCI performance and usability (von Groll et al., 2024; Fry et al., 2022; Kögel et al., 2020; Ahn and Jun, 2015). All these considerations are critical for translating BCI-based ARTs from laboratory to commercial-grade use, providing widespread access to people with disabilities.

### 1.1 Scope of the review

This narrative review provides a broad yet critical evaluation of the state-of-the-art BCI-based ARTs, employing a flexible method for reviewing and selecting the literature. Research databases, including CINAHL, Embase, Engineering Village, IEEE Xplore Digital Library, MEDLINE, and PubMed, were utilized to search for relevant literature. Publications addressing the current state of BCI-based ARTs were selected and included in this review. Search terms were used related to: (1) individualization of BCI; (2) user-centric design strategies for ARTs; and (3) to fulfil the evolving functional and developmental needs of users with disabilities.

This review contributes to the field by providing a holistic overview of BCI-based ARTs in terms of user-centric design philosophy to fulfil individualized user requirements of people with disabilities. Studies have investigated the importance of an inclusive, personalized approach for the development of communication BCI for children and adults with disabilities, not only due to inter-individual variability, but also due to the importance of including opinions from all stakeholders (Shrivastava et al., 2025; Branco et al., 2025; Russo et al., 2025; French et al., 2024; Ivanov et al., 2023; Branco et al., 2023). (Edelman et al. 2024) have recently evaluated current advancements in noninvasive BCI technologies. At the same time, other reviews have focused on invasive options (Dunlap et al., 2020; Ferguson et al., 2019). In contrast, this review compares both invasive and noninvasive neuroimaging techniques for their suitability in diverse ARTs for people with disabilities, with a perspective on user needs. (Patrick-Krueger et al. 2024) have explored the current state of clinical trials of implantable BCIs. Some reviews have investigated different digital signal processing, machine learning, and artificial intelligence techniques, as well as BCI decoding algorithms (Saha and Baumert, 2020; Azab et al., 2018; Lotte et al., 2018; Jayaram et al., 2016; Krusienski et al., 2011; Bashashati et al., 2007; Lotte et al., 2007). (Saha et al. 2021) published a review on BCI advancements, discussing the diverse application areas of BCI in general, unlike this review, which addresses individualized applications for people with disabilities. On the contrary, most, if not all, state-of-the-art reviews aim to consider a particular user group or a specific challenge or opportunity in terms of neuroimaging, signal processing, pattern recognition, clinical and socioeconomic aspects of BCI development (Patrick-Krueger et al., 2024; Edelman et al., 2024; Bergeron et al., 2023; Kaongoen et al., 2023; Karikari and Koshechkin, 2023; Lopez-Bernal et al., 2022; Karlsson et al., 2022; Keser et al., 2022; Fry et al., 2022; Gao et al., 2022; Hramov et al., 2021; Mrachacz-Kersting et al., 2021; Orlandi et al., 2021; Dunlap et al., 2020; Duun-Henriksen et al., 2020; Martini et al., 2020; Micera et al., 2020; Saha and Baumert, 2020; Ferguson et al., 2019; Azab et al., 2018; Deffieux et al., 2018; Lotte et al., 2018; Jayaram et al., 2016; Klein and Ojemann, 2016; Krusienski et al., 2011; Bashashati et al., 2007; Lotte et al., 2007; Dobkin, 2007).

## 2 Brain-computer interface for people with disabilities

**Figure 2:**
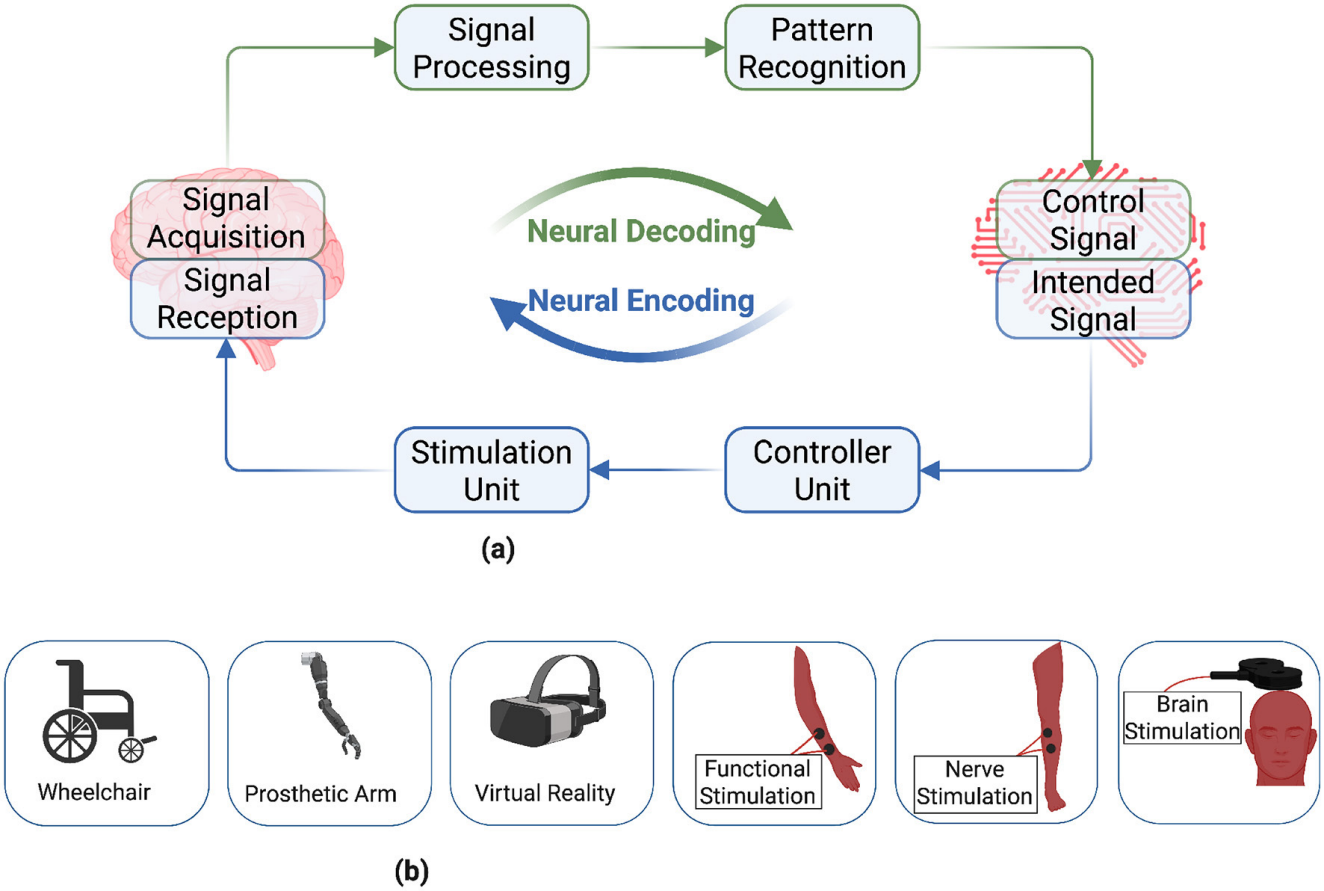
Block diagram of a bidirectional brain-computer interface (BCI) with peripheral assistive and rehabilitative technologies (ARTs). A BCI enables the decoding and encoding of neural information from and into the brain, and can be used to operate different ARTs. Diversity in impairments and disabilities for people with disabilities leads to highly individualized user needs that demand user-centric design of BCI-based ARTs. **(a)** Brain computer interface. **(b)** Assistive and Rehabilitative technologies. Created in BioRender (https://BioRender.com/496j0pt) licensed under CC BY 4.0.

A BCI facilitates functional interaction between the brain and a computer, enabling the decoding and encoding of neural information from and into the brain (Card et al., 2024; Willett et al., 2023; Huggins et al., 2022; Karlsson et al., 2022; Orlandi et al., 2021; Mrachacz-Kersting et al., 2021; Willett et al., 2021; Saha et al., 2021; Moses et al., 2021; Flesher et al., 2021; Mrachacz-Kersting et al., 2016; Rao et al., 2014; Moghimi et al., 2013). It provides novel ways of communication for end-users intending to interact with their surroundings through ARTs. Figure 2 illustrates a simplified block diagram of a bidirectional BCI and its application in diverse ARTs for people with disabilities. Neural decoding utilizes implantable or non-implantable neuroimaging modalities to record electrical and hemodynamic responses of the brain that corresponds to a user's intentions or cognitive states (Liu et al., 2025; Chen et al., 2025; Saha et al., 2021; Moghimi et al., 2013; Patrick-Krueger et al., 2024; Edelman et al., 2024; Karikari and Koshechkin, 2023; Martini et al., 2020; Min et al., 2010). The captured brain signals are classified through digital signal processing and pattern recognition algorithms (Lotte et al., 2018; Krusienski et al., 2011; Bashashati et al., 2007; Lotte et al., 2007) to use them to operate different types of ARTs, for example, wheelchairs, prosthetic arms, virtual reality, and neurostimulation modalities (Mrachacz-Kersting et al., 2021; Saha et al., 2021; Flesher et al., 2021; Mrachacz-Kersting et al., 2016; Cao et al., 2014).

Other assistive technologies, such as eye-tracking and voice-controlled devices (Sunny et al., 2021; Tran et al., 2020), may also offer assistance for users than BCI-based ARTs. However, there are circumstances when BCI outperforms other existing ART interfaces. A BCI typically exploits residual brain functions explicitly without any peripheral muscular input, and this is critical for people with severe disabilities post-neurological incidents (Willett et al., 2023; Moses et al., 2021). Users with limited or no functional abilities may not operate an eye-tracking or voice-controlled device. By utilizing residual brain functions, BCI-based ARTs can provide effective rehabilitation interventions or alternative communication links for users to interact with their surroundings. Thus, the appropriateness of BCI-based ARTs highly depends on individual circumstances. Nonetheless, the rapid evolution of BCI technologies in recent years makes them suitable for manifold applications for diverse user cohorts (Edelman et al., 2024; Karikari and Koshechkin, 2023; Lopez-Bernal et al., 2022; Karlsson et al., 2022; Saha et al., 2021). Table 1 summarizes some state-of-the-art original research studies on BCI-based ARTs for people with diverse types of disabilities.

**Table 1:** Key studies on brain computer interface (BCI)-based assistive and rehabilitative technologies (ARTs) for people with disabilities.

**References**	**User group**	**Disability addressed**	**Signal type**	**Key contribution**
**Branco et al. (2025)**	**Parents and caregivers of children or young adults with cerebral palsy**	**Gross motor function classification system scores (I–V)**	**Motor or speech imagination and visual or auditory evoked potential**	**Emphasizing a personalized approach for the development of communication BCI for children and young adult with quadrepgic cerebral palsy due to inter-subject variable characteristics**
Brunner et al. (2024)	Stroke	Upper limb paralysis	Motor imagery	BCI integrating functional electrical stimulation for post-stroke rehabilitation
Card et al. (2024)	Amyotrophic lateral sclerosis	Tetraplegia and dysarthria	Attempted speech	A high-performance speech neuroprosthesis
Soldado-Magraner et al. (2024)	Various	Various	Various	IEEE Neuroethics Framework, an international, multiyear, iterative initiative for diverse BCI stakeholders
Lorach et al. (2023)	Spinal cord injury	Tetraplegia	Attempted movement	A bidirectional brain–spine interface helping to walk naturally
Metzger et al. (2023)	Brainstem stroke	Limb and vocal tract paralysis	Attempted speech	A high-performance speech BCI and avatar control featuring advanced deep learning algorithms
Mitchell et al. (2023)	Amyotrophic lateral sclerosis	Upper limb paralysis	Attempted movement	A safety study of minimally invasive strentrode-based BCI
Rubin et al. (2023)	Spinal cord injury and brainstem stroke, etc.	Quadriplegia	Intended movement	Comparable safety profile of BrainGate Neural Interface system with other chronically implanted medical devices
Willett et al. (2023)	Amyotrophic lateral sclerosis	Speech	Attempted speech	An alternative naturalistic speech communication pathway for a person with paralysis
Branco et al. (2023)	Locked-in syndrome	Severe speech and physical	Motor or speech imagination and visual or auditory evoked potential	Identifying user preferences for active communication BCI (e.g., motor or speech imagery) over reactive BCIs such as visual or auditory evoked potential
Chaudhary et al. (2022)	Amyotrophic lateral sclerosis	Completely locked-in state	Auditory evoked potential	A communication BCI for a person with completely locked-in state
Jadavji et al. (2022)	Cerebral palsy	Severe speech and physical	Motor imagery	Assisting children with cerebral palsy through patient-centered clinical BCI for communication and play
Flesher et al. (2021)	Spinal cord injury	Tetraplegia	Intended movement	Microstimulation-based bidirectional BCI improves robotic arm control
Jovanovic et al. (2021)	Spinal cord injury	Paretic upper extremities	Attempted movement	Illustrating a BCI-driven functional electrical stimulation as safe, feasible and promising rehabilitation
Woeppel et al. (2021)	Spinal cord injury and brainstem stroke, etc.	Tetraplegia	Sensorimotor rhythm	Clinical trials on safety profiles of the Utah array and their explantation for maintaining chronic recording of neural signals
Kasahara et al. (2018)	Parkinson's disease	Tremor, stiffness and slowing of movement, etc.	Motor imagery	A pilot study for a user to use BCI with and without antiparkinsonian medication
Andresen et al. (2016)	Locked-in syndrome	Severe speech and physical	Rapid Serial Visual Presentation	Proposition of patient-centered outcome measures to evaluate BCIs
Vansteensel et al. (2016)	Amyotrophic lateral sclerosis	Severe paralysis	Attempted movement	A BCI complementing or often outperforming eye tracking device for communication

### 2.1 Necessity of user-centric design

The practical usability of a BCI depends on various factors, primarily the user-centric design of ARTs (Shrivastava et al., 2025; Russo et al., 2025; Branco et al., 2025; French et al., 2024; Ivanov et al., 2023; Kübler et al., 2014). The user-centric design is an iterative approach that includes users' perspectives while developing BCI-based ARTs to fulfil individualized user needs. The ICF framework aligns closely with user-centric design principles in BCI development. By recognizing the influence of environmental and personal factors, such as caregiver support, living conditions, and user preferences, the ICF supports the creation of adaptable technologies that reflect real-world diversity in user needs and contexts. It provides a holistic method to evaluate the usability of ARTs for people with disabilities. Some studies proposed user-centric design-based techniques for assessing usability of BCI applications in real-life settings, going beyond developer-centric evaluation criteria such as classification accuracy and information transfer rate (Ivanov et al., 2023; Kübler et al., 2014). Other studies conducted surveys on people with disabilities and their caregivers seeking their opinions on BCI-based ARTs (Shrivastava et al., 2025; Russo et al., 2025). Overall, BCIs are acceptable; however, the usability will depend on how developers integrate the input from the users in the development of BCI-based ARTs.

The clinical distinction of neurological variability defines disability types and promotes individualization of BCI-based ARTs. While one group of users encounters developmental disabilities like people with cerebral palsy, others experience acquired disabilities due to neurological incidents such as stroke, amyotrophic lateral sclerosis, and spinal cord injury (Metzger et al., 2023; Bekteshi et al., 2023; Hallett et al., 2022; Monforte et al., 2021; Makris et al., 2021; Micera et al., 2020; Goulet et al., 2019; Milekovic et al., 2018; Goldstein and Abrahams, 2013). Due to the diverse types and severities of impairments that reflect individualized user needs (Seghier and Price, 2018; Park et al., 2016), the user-centric and personalized design of BCI-based ARTs may better fulfil the individualized requirements of people with disabilities (French et al., 2024; Branco et al., 2023; Saha et al., 2021; Martin et al., 2018).

### 2.2 Assistive and rehabilitative technologies

The principles of BCI-based assistive or communication technologies rely on how brain signals are translated into machine commands, for example, using two distinct brain activity patterns to control a switch (on/off) (Cao et al., 2014; Mitchell et al., 2023). These can take active, reactive and passive forms of translation through a BCI. The active BCI translates users' intentions, such as imagined movements and speech, into control signals or messages on a computer screen utilizing sophisticated decoding algorithms and software (Willett et al., 2023, 2021; Saha et al., 2021; Moses et al., 2021; Acı et al., 2019; Aricò et al., 2018; Zander et al., 2009). However, the success of these applications relies on how accurately the decoding algorithms can classify brain activities (Lotte et al., 2018; Krusienski et al., 2011; Bashashati et al., 2007; Lotte et al., 2007). Visual and auditory evoked potentials are signals due to external stimulation and define reactive BCI applications. Reactive BCIs are suitable for people who have severe disabilities, for example, individuals in locked-in states (Chaudhary et al., 2022). Depending on the disability type and severity, the specific signal type is selected to meet individualized user needs. For example, a person with a visual impairment is not likely to use a visually evoked potential-based BCI but may find an auditory cue-based stimulation more useful. While reactive BCIs are time-synchronized to external stimuli, active ones offer intuitive use for functional autonomy. For visually evoked potential, studies found that not all individuals with disabilities perform equally for varied types of external visual stimulation, e.g., steady-state visual evoked potential vs. P300, and checkerboard flashing vs. row-column flashing-based BCIs (Severens et al., 2014; Combaz et al., 2013; Townsend et al., 2010). Thus, a BCI system is unlikely to work well for all users, and poor performance with one system does not mean no system would work for that user. Finally, a passive BCI is applicable when monitoring users' affective states, such as detecting drowsiness. There is no one-size-fits-all; nonetheless, BCI-based ARTs require assessments of users' perspectives of gaining functional autonomy and codesign to best fulfil individualized needs (French et al., 2024; Huggins et al., 2015; Nijboer et al., 2014; Huggins et al., 2011). Individualized BCI design can be guided by the ICF's emphasis on activity limitations and participation restrictions. For example, a user's ability to engage in daily tasks or social roles may be constrained by both impairments and contextual barriers, which BCI-based ARTs can help overcome.

The principles of rehabilitative BCIs rely on promoting neuroplasticity, which refers to the adaptive behavior of central and peripheral neural networks. Neuroplasticity is a critical ingredient of the motor learning process and is at the center of BCI-driven rehabilitation (Flesher et al., 2021; Gao et al., 2022; Saha and Baumert, 2020; Dobkin, 2007). Neurostimulation modalities are the main components of neural encoding that externally modulate neural activities in target brain areas or peripheral neural networks to repair paretic functional abilities by rendering neuroplasticity (Flesher et al., 2021; Mrachacz-Kersting et al., 2016). They are feedback elements of some closed loop or open loop rehabilitative BCIs, mostly restimulating impacted neural networks in the brain and central and peripheral nervous systems. Studies proposed BCI-based rehabilitation strategies featuring transcranial magnetic stimulation and direct/alternating current stimulation for repairing stroke lesions in the brain (Keser et al., 2022; Van der Groen et al., 2022; Yang et al., 2021; Antal and Herrmann, 2016). Some implantable options include deep brain stimulation and intracortical microstimulation (Allert et al., 2018; Willett et al., 2023, 2021; Oxley et al., 2016). While the primary applications of neurostimulation techniques are in neurorehabilitation, they can also be used to improve signal quality and users' ability to operate a BCI (Won et al., 2023). (Lorach et al. 2023) developed a rehabilitative BCI featuring epidural nerve stimulation for rectifying spinal cord injury. Other studies presented BCI-driven functional electrical stimulation for rehabilitating impaired upper or lower limb functions (Brunner et al., 2024; Jovanovic et al., 2021; Mrachacz-Kersting et al., 2021, 2016). BCI-driven neurofeedback has been reported to regulate the cortical-subcortical networks and assists in modulating brain signals for cognitive or functional recovery (Silversmith et al., 2021; Sitaram et al., 2017; Orsborn et al., 2014). Notably, both neurofeedback and neurostimulation may involve decoding users' intentions from brain activities to operate ARTs. Neurostimulation is an essential element of a bidirectional BCI, specifically for neural encoding; however, a more detailed investigation on neurostimulation is beyond the scope of this review, and the subsequent sections will emphasize neuroimaging modalities.

## 3 Advances in BCI technologies

### 3.1 Implantable vs. non-implantable neuroimaging techniques

Neuroimaging is a critical element of neural decoding, which defines the appropriateness of BCI-based ARTs across diverse user groups with different types of disabilities (Patrick-Krueger et al., 2024; Edelman et al., 2024; Karikari and Koshechkin, 2023; Moghimi et al., 2013). Typically, no single modality offers the spatial, spectral and temporal resolution for mapping all required complex brain functions, resulting in limited BCI control signals (Saha et al., 2021; Martini et al., 2020; Min et al., 2010). However, recently developed implantable speech BCIs have demonstrated up to 50-word decoding and constructing thousands of sentences by integrating language models (Willett et al., 2023, 2021; Moses et al., 2021). Electroencephalography (EEG), magnetoencephalography (MEG), electrocorticography (ECoG) and microelectrode arrays (MEA) capture fine temporal features (i.e., electrical activities). However, the sensors are either sparsely distributed (spatially) or localized within an area of interest only. Functional magnetic resonance imaging (fMRI) records high-resolution spatial features of the brain, but the unmanageable size and slow hemodynamic signals are unsuitable for many real-time applications (Sitaram et al., 2007). As an alternative, functional near-infrared spectroscopy (fNIRS) has lower spatial resolution but is more practical, mainly due to its portability and ease of use (Naseer and Hong, 2015). On the other hand, electric and magnetic field-based neuroimaging techniques, i.e., EEG and MEG, offer better speed and bandwidth. MEG lacks portability and is barely usable outside a specialized setting (Bu et al., 2023). Table 2 illustrates the key characteristics of the neuroimaging modalities and their applicability in BCI-based ARTs (Saha et al., 2021; Hramov et al., 2021; Deffieux et al., 2018; Nicolas-Alonso and Gomez-Gil, 2012; Min et al., 2010).

**Table 2:** Key characteristics of neuroimaging techniques and their applicability in BCI applications.

**Feature**	**EEG**	**MEG**	**ECoG**	**MEA**	**fMRI**	**fNIRS**
Signal type	Electrical	Magnetic	Electrical	Electrical	Metabolic	Metabolic
Measurement type	Direct	Direct	Direct	Direct	Indirect	Indirect
Implantable	No	No	Yes	Yes	No	No
Portable	Yes	No	Yes	Yes	No	Yes
Temporal resolution	~0.05 s	~0.05 s	~0.003 s	~0.003 s	~1 s	~1 s
Spatial resolution	~10 mm	~5 mm	~1 mm	~0.05–0.5 mm	~1 mm	~5 mm
Brain coverage	Specified by varying electrode numbers and their placements, can spread across the scalp covering different areas of the brain	Specified by varying sensor numbers and their placements, can cover different areas of the brain	Varying number of high-density electrodes, but typically localized to capture specific brain activities	Varying number of very high-density electrodes to capture local brain activities	Volumetric imaging capturing near real-time functional brain activities by reconstructing whole head anatomy	Specified by varying sensor numbers and their placements, can cover different areas of the brain
BCI aplicability	High due to no surgical needs, low cost, easy maintenance, portability and very good temporal signal for real-time BCI	Very good temporal signals for real-time BCI applications like EEG, but limited by high cost, lack of portability and requirement of specialized setup	Appropriate for people with disabilities when benefits outweigh risk factors, but unfavourable for healthy users due to surgical risk factors and post-implantation maintenance	Appropriate for people with disabilities when benefits outweigh risk factors, but unfavourable for healthy users due to surgical risk factors and post-implantation maintenance	Impractical due to slow signal and mobility constraint, but good for source localization	Slow signal, but practical alternative to fMRI due to portability

Saha et al. (2021), Hramov et al. (2021), Deffieux et al. (2018), Nicolas-Alonso and Gomez-Gil (2012), and Min et al. (2010).

BCI, brain-computer interface; EEG, electroencephalography; MEG, magnetoencephalography; ECoG, electrocorticogram; MEA, microelectrode array; fMRI, functional magnetic resonance imaging; fNIRS, functional near infrared spectroscopy.

**Figure 3:**
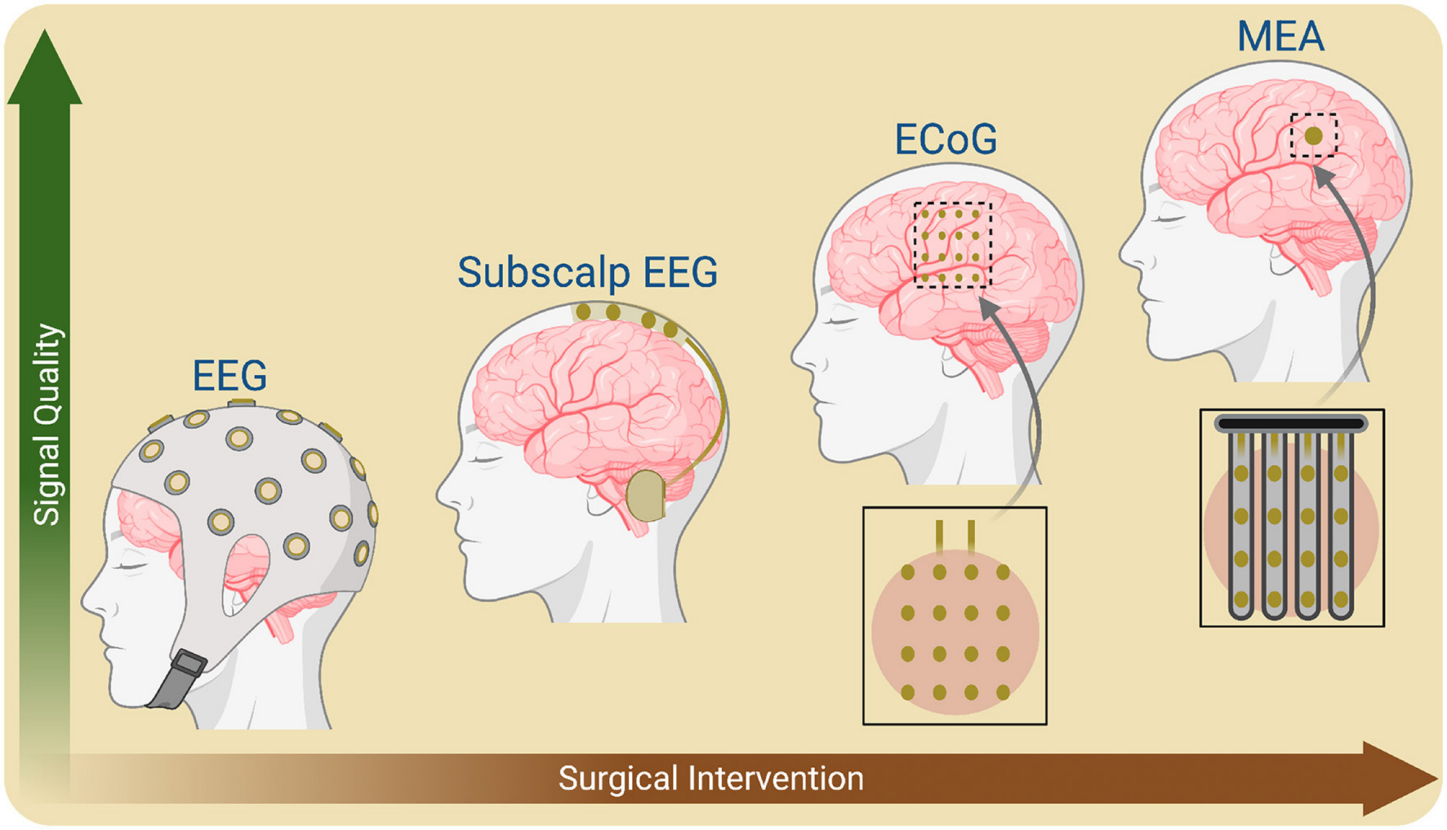
A schematic illustration of signal quality and surgical intervention of neuroimaging modalities to record the brain's electrical activities. Implantable options, such as microelectrode arrays (MEA) and electrocorticography (ECoG), offer superior signal quality at the cost of increased surgical risk factors, compared to safer and noninvasive methods, including electroencephalography (EEG) and magnetoencephalography (MEG). Created in BioRender (https://BioRender.com/g514fz2) licensed under CC BY 4.0.

From a translational perspective, EEG is still one of the most viable neuroimaging modalities due to its portability, easy maintenance and low cost. It does not involve any surgical procedure, like a craniotomy for implantable ECoG and MEA. Moreover, EEG could enable wearable BCIs using tiny recording setups like ear EEG (Kaongoen et al., 2023). However, EEG signals are highly nonstationary and nonlinear due to time-variant and subject-specific anatomical, psychological, physiological, and environmental factors (Saha et al., 2023, 2021; Saha and Baumert, 2020; Saha et al., 2019, 2017a,b). Furthermore, EEG signals do not offer the required spatial resolution for mapping localized brain functions because the signals attenuate through the outer brain layers, skull, scalp, skin and hairs (Miinalainen et al., 2019; Cohen, 2017). This issue is somewhat alleviated with implantable ECoG and MEA, which record functionally relevant localized signals (Vansteensel et al., 2016). However, the higher risk associated with craniotomy diminishes the utility of these neuroimaging modalities. For example, in cases where the long-term trajectory of user benefits and associated risk factors remains unclear to stakeholders. There are implanted alternatives that use less extensive surgery, such as Stentrode, a new type of neuroimaging introduced by Synchron^TM^ (Oxley et al., 2016). This system does not require craniotomy. Instead, it is computer-guided, with a minimally invasive procedure used to guide the Stentrode into a blood vessel within the brain. Studies have found that the signals are comparable to their counterparts with more extensive surgery, such as ECoG and MEA. (Mitchell et al. 2023) discovered Stentrode to be a safe implantable endovascular BCI for people with severe paralysis. However, a limitation of Stentrode is that it records brain signals from only the major blood vessels; thus, a question remains if this technology is scalable to any brain area. Sub-scalp EEG has recently become another viable option for recording finer resolution signals than EEG and requires less maintenance than ECoG and MEA (Duun-Henriksen et al., 2020). Generally, signal quality tends to improve with increasing extensive surgery in current neuroimaging modalities of BCI (Figure 3). For example, the invasive Utah intracortical eletrode array and minimally invasive stentrode offer significantly superior signal quality as compared to EEG and is suitable for diverse BCI-based ARTs (Mitchell et al., 2023; Grani et al., 2022; Oxley et al., 2016; Maynard et al., 1997).

#### 3.1.1 Implantation lifespan and brain development

The appropriateness of a neuroimaging modality requires careful consideration, weighing up risks and benefits (Rubin et al., 2023; Leuthardt et al., 2021; Sierra-Mercado et al., 2019), especially in cases of implantable BCI-based ARTs for people of different ages with diverse disabilities. Current implantable electrodes are increasingly biocompatible, provide good-quality signals for up to several years, and hold a promise to operate a BCI successfully. However, findings show that the neural signal quality deteriorates over time due to implant-related factors such as tissue encapsulation and material degradation (Woeppel et al., 2021; Ferguson et al., 2019), and reimplanting sensors is a complex task due to surgical risk factors (Sponheim et al., 2021; Viana et al., 2021; Woeppel et al., 2021; Sierra-Mercado et al., 2019). The necessity of reimplantation and post-operative maintenance are critical aspects of implantable BCIs over the lifespan of a user. An implantable BCI is often permanent, and the device is kept inside the brain due to anatomical changes in complex brain structure and risk factors associated with surgical extraction before potential reimplantation (Fry et al., 2023). A question remains whether the current implantable BCI can offer long-term use while minimizing the chances of reimplantation. Further advances in neural sensor technologies, decoding algorithms and computing platforms in miniaturized format may lengthen the lifespan of implantable BCIs (Shaeri et al., 2024; Steinmetz et al., 2021). However, more studies investigating the implant lifespan and safety considerations of implantation and reimplantation are essential to substantiate the usability of implantable BCI-based ARTs for people with disabilities.

**Figure 4:**
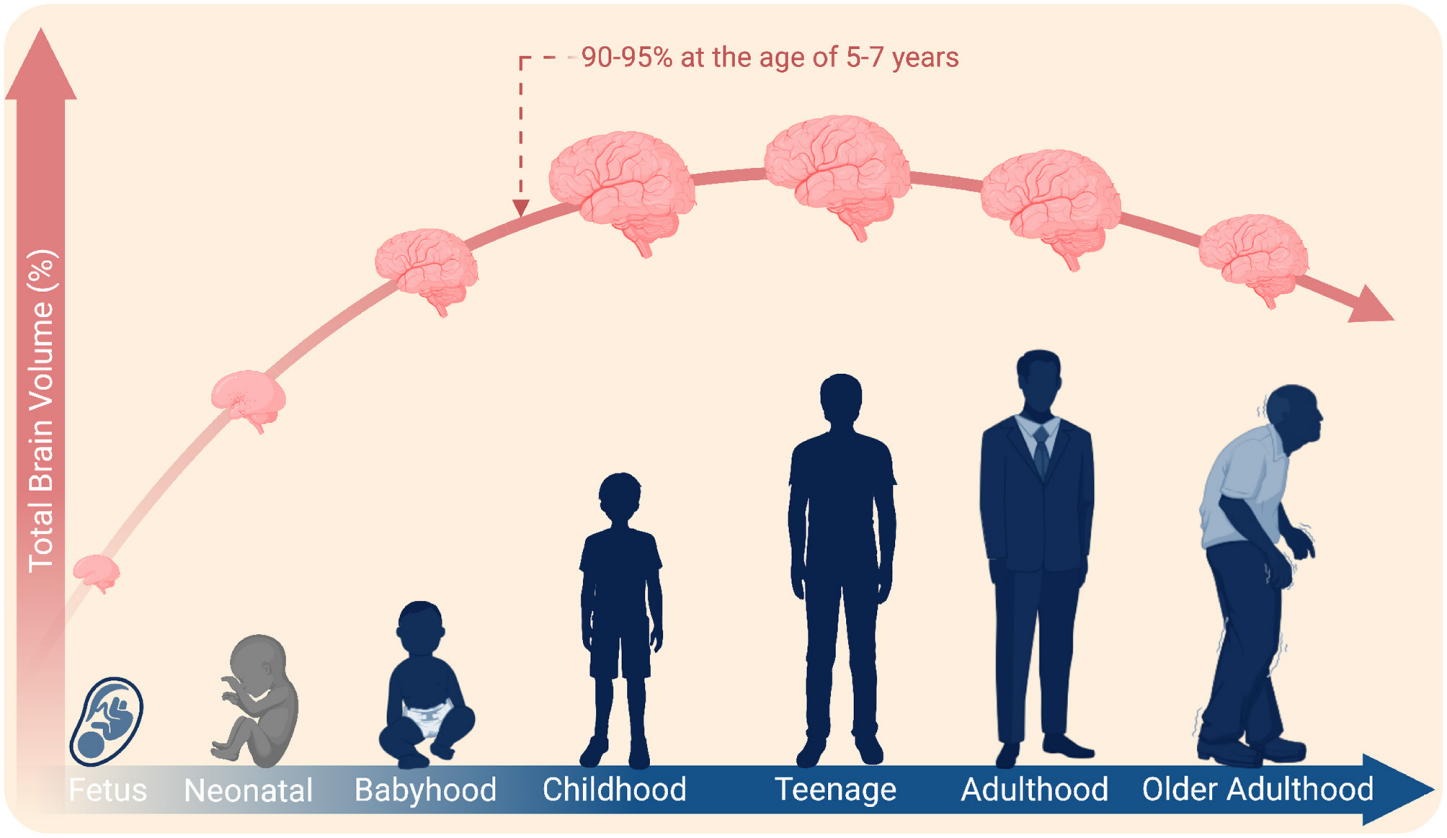
A schematic timeline illustrating changes in total brain volume: 90%–95% growth occurs within the age of 5–7 years. Created in BioRender (https://BioRender.com/qlb8yew) licensed under CC BY 4.0.

In pediatric BCI, where the benefits of early implantation might take advantage of neurodevelopment and plasticity, the changing environment around implanted sensors over the lifespan is another essential aspect. Around 90%–95% of human brain growth occurs by the age of 5–7 years, although cognitive experiences evolve over the lifetime (Zhou et al., 2024; Bethlehem et al., 2022; Hedman et al., 2012; Brown and Jernigan, 2012; Peters, 2006) (Figure 4). Non-implantable neuroimaging, specifically EEG, offers BCI-based ARTs for pediatric users living with movement and speech disabilities (Kelly et al., 2023; Jadavji et al., 2022). However, there is a lack of studies to substantiate the potential use of implantable BCI for pediatric users. The human brain undergoes anatomical transformations during its lifespan; for example, it expands and shrinks in the early and late ages (Bethlehem et al., 2022). Moreover, electrode displacements post-implantation may contribute to increased chances of reimplantation (Göransson et al., 2021; Morishita et al., 2017; Lumsden et al., 2015). As neural technologies advance and growing evidence are accessible for researchers and clinicians, the BCI-based ARTs may soon evolve in children with severe disabilities after careful technical and ethical considerations (Bergeron et al., 2023). Although initially assumed inappropriate, cochlear implant is a notable example that now benefit pediatric users and demonstrate the promise of implantable neurotechnologies for people of all ages (Chennareddy et al., 2025). Other examples of implantable neurotechnologies, though not mainstream, include responsive neurostimulation to reduce the dominance of epileptic seizures and deep brain stimulation to treat dystonia-related motor symptoms and disabilities (Hartnett et al., 2022; Hale et al., 2020). In some cases, non-implantable BCIs tend to be more appropriate, for example, EEG-based BCIs for the elderly (Belkacem et al., 2020).

### 3.2 Neural signal processing and pattern recognition

The most advanced BCIs utilize the brain's electrical activities recorded through neuroimaging techniques such as EEG, ECoG and MEA (Edelman et al., 2024; Willett et al., 2023, 2021; Moses et al., 2021; Sponheim et al., 2021; Saha et al., 2021; Edelman et al., 2019; Oxley et al., 2016; Rao et al., 2014; LaFleur et al., 2013). Other state-of-the-art modalities include MEG to measure magnetic fields produced by the brain's electrical currents and fNIRS to record the brain's hemodynamic activities (Bu et al., 2023; Naseer and Hong, 2015). These methods output time-domain signals corresponding to the user's intentions or cognitive states. Traditionally, various digital signal processing techniques filter out undesired noises or artefacts from raw neural signals to enhance the detectability of user's intentions or cognitive states (Lotte et al., 2018; Krusienski et al., 2011; Bashashati et al., 2007; Lotte et al., 2007). Then, handcrafted feature extraction follows a classifier to evaluate the decoding accuracy of a BCI. However, with the recent developments in artificial intelligence-based algorithms, nonlinear activation functions replace handcrafted feature engineering for attributing more generic features associated with users' intentions or cognitive states (Barmpas et al., 2023; Roy et al., 2020; Fahimi et al., 2019). Convolutional neural network (CNN)-based architectures are popular among the state-of-the-art artificial intelligence techniques. The raw neural signals may require preprocessing to enhance signal quality and transformation to represent the signals as compatible with CNN or other architectures (Kamble et al., 2023a,b; Garćıa-Salinas et al., 2023). For example, raw neural signals are directly compatible as input to a 1-dimensional CNN or long short-term memory network architecture; however, the time-domain signals are incompatible as input to 2-dimensional CNN architectures. In this case, converting time-domain neural signals into 2-dimensional images through time-frequency analyses such as short-time Fourier transform and wavelet transform is typically performed.

Recent advancements in BCI technologies have significantly improved their performance. An implantable MEA has demonstrated an excellent 94% decoding accuracy for a 50-word classification task for a speech neuroprosthesis (Willett et al., 2023). Non-surgical EEG systems, while less precise, show around 60% average accuracy for decoding up to 6 words (Bhalerao and Pachori, 2025). A non-implantable EEG-based speller BCI can also provide high accuracy, such as 82% average accuracy, featuring visually evoked potential and few-shot learning (Miao et al., 2024). However, decoding using the non-surgical EEG-based speller paradigm is much slower than word decoding by implantable BCIs. The trade-off between speed and accuracy is important while selecting an appropriate BCI type for a user, by considering the disability and user-centric technical specifications fulfilling individualized user needs and preferences. Although surgical options offer superior performance to non-surgical BCIs, surgical options are highly individualized and tailored to demonstrate user-specific high decoding accuracy. On the contrary, EEG signals are more accessible to many users with advanced decoding algorithms, some of which are generalizable across users.

The time-domain neural signals fluctuate over time and across users due to diversity in anatomical, physiological, psychological and cognitive characteristics (Saha and Baumert, 2020). These inter-session and inter-subject variabilities cause differences in training and testing feature distributions, leading to covariate shifts. Covariate shift occurs when training and testing feature domains differ due to inherent variability in data characteristics, resulting in poor decoding accuracy (Saha and Baumert, 2020; Azab et al., 2019, 2018; Jayaram et al., 2016). For covariate shift adaptation, a BCI typically requires tedious calibration using data from a new target subject or the same subject on a new session. Studies demonstrated significant inter-session and inter-subject variabilities, even in healthy user cohorts (Saha et al., 2019, 2017b; Kang and Choi, 2014; Kang et al., 2009). The impact of these variabilities should be more prominent for users with disabilities due to the diversity in impairments post-neurological incidents (Park et al., 2016).

Studies proposed various transfer learning strategies in BCI decoding algorithms for covariate shift adaptation (Azab et al., 2019, 2018; Jayaram et al., 2016). Inter-session and inter-subject domain adaptation is an example of transfer learning in BCI. However, most transfer learning techniques require at least a few training data for decoding algorithm calibration while the goal is a fully zero-training BCI that promotes wide dissemination of this technology across diverse user groups. Inter-subject associative BCI is feasible requiring no training data from the target user when the training and testing subjects share similar brain dynamics (Saha et al., 2023, 2019, 2017a,b). Quantifying inter-subject associativity is a way to predict the performance of fully zero-training BCI. From a translational perspective, generalized decoding algorithms can disseminate BCI-based ARTs to a large cohort by minimizing the calibration requirements (Roy et al., 2020; Saha et al., 2017b). Even so, a large cohort of people (15%–30%) encounter BCI deficiency, which refers to BCI system's inability to interpret users' brain signals (Park and Jun, 2024; Bamdadian et al., 2015; Vidaurre and Blankertz, 2010).

## 4 Translational socioeconomic outlook of BCI technologies

The ICF framework provides a valuable foundation for policy and regulatory development. Its standardized language for describing functioning and disability can facilitate cross-sector communication among clinicians, engineers, policymakers, and users, supporting ethical and inclusive deployment of BCI technologies. BCI-based ARTs can assist people with disabilities in accessing mainstream socioeconomic life, where they can contribute to the ethical and economic dynamics of society. The ICF framework supports inclusive policy development by highlighting contextual factors affecting participation across all health conditions. Regardless of scientific breakthroughs, users' perspectives on using BCI-based ARTs and their safety are critical (Patrick-Krueger et al., 2024; Soldado-Magraner et al., 2024; Bergeron et al., 2023; Saha et al., 2021; Naufel and Klein, 2020; Sample et al., 2019; Klein and Ojemann, 2016; Nijboer et al., 2013). Individualization of BCI-based ARTs by user-centric design strategy is vital where users can participate in policy-making and take informed decisions on their preferences, comforts and interaction with the technologies (Shrivastava et al., 2025; Branco et al., 2025). While all stakeholders must ensure the ethical use of BCI, users with disabilities and their caregivers should be at the center of research, development, and policies for maximizing the benefits of advanced BCIs. Informed consent from the users or their caregivers is the precursor to using a BCI after adequately making the user aware of the risk factors and the expected benefits. Some users with disabilities may experience cognitive or communication difficulties; thus, ensuring they are fully informed about risks and benefits may be challenging (Willett et al., 2023, 2021; Moses et al., 2021; Vansteensel et al., 2016). Consent may be challenging in children (Bergeron et al., 2023); and careful consideration is required as their perspective of using a BCI may change by the time they reach adulthood. They may question their parent's or guardian's decision on whether to implant a BCI in their early years. Studies underlined the importance of engaging BCI stakeholders considering ethics policies to ensure transparent communication with users before obtaining consent (Patrick-Krueger et al., 2024; Soldado-Magraner et al., 2024; Bergeron et al., 2023; Saha et al., 2021; Naufel and Klein, 2020; Sample et al., 2019).

Standardizing BCI-based ARTs and their lawful utilization is crucial for positive societal change (Patrick-Krueger et al., 2024; Soldado-Magraner et al., 2024; Bergeron et al., 2023; Chandler et al., 2022). BCI is an emerging field, and authorities should act promptly to set up regulations that include all stakeholders' opinions due to the fast evolution of BCI-based ARTs in diverse areas. The Brain/Neural Computer Interaction Horizon 2020 project defined six primary objectives of BCI: (1) restore (e.g., unlocking the residual ability of completely locked-in); (2) replace (e.g., BCI-based ARTs); (3) enhance (e.g., improved user experience in video games); (4) supplement (e.g., interactive virtual/augmented/mixed reality glasses); (5) improve (e.g., upper/lower limb rehabilitation post stroke); and (6) research tools (e.g., decoding brain activity with real-time neurofeedback) (Brunner et al., 2015). The development of regulatory guidelines may address the socioeconomic concerns and reflect the defined objectives of BCIs in these application areas. (Patrick-Krueger et al. 2024) discussed the clinical perspectives of implantable BCIs and the importance of regulatory approvals after clinical trials of the state-of-the-art technologies. A question remains whether BCI guidelines could limit their use after carefully evaluating the risks vs. benefits and socioeconomic impact trajectories (Patrick-Krueger et al., 2024; Soldado-Magraner et al., 2024; Coin and Dubljević, 2023; Sample et al., 2019).

**Figure 5:**
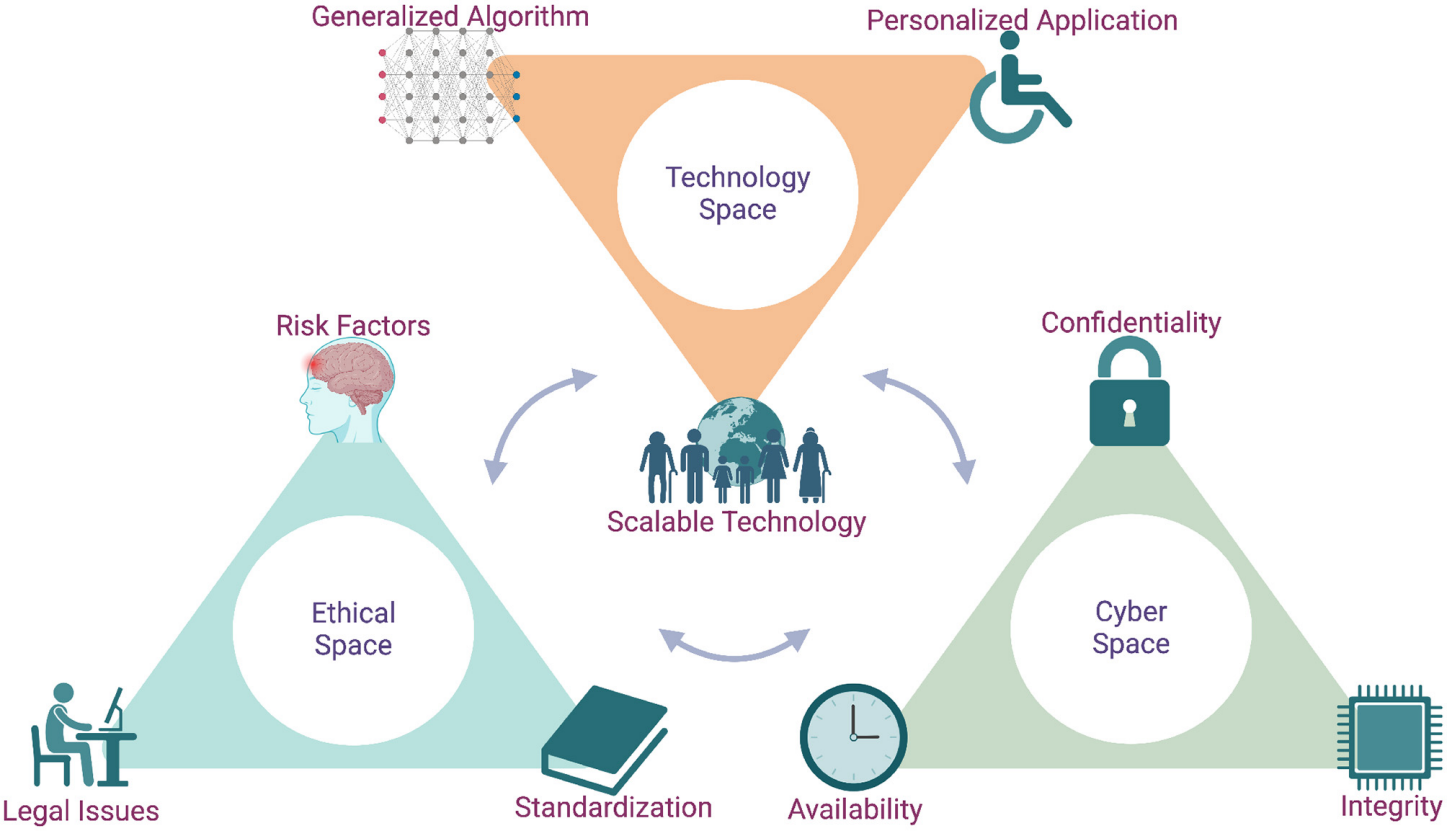
The translational outlook of brain-computer interface (BCI)-based assistive and rehabilitative technologies (ARTs). The sustainable development of BCI-based ARTs that can benefit people with disabilities depends on manifold translational considerations in technology, ethical and cybersecurity spaces. Individualized design can fulfil user needs, but a generalized algorithm can improve the scalability of BCI for widespread dissemination across users. However, ethical considerations such as surgical and non-surgical risk factors, technology standardization, lawful utilization, and cybersecurity measures like preventing unauthorized neural data access are equally critical translational aspects of BCI-based ARTs. Created in BioRender (https://BioRender.com/i3tkkml) licensed under CC BY 4.0.

Emerging cybersecurity measures are also essential translational elements of BCI-based ARTs. Confidentiality, integrity and availability are critical cybersecurity components to ensure ethical BCI use (Liv and Greenbaum, 2023; Bernal et al., 2023). Confidentiality prevents unauthorized access to sensitive neural data, integrity ensures data precision, and availability maintains the infrastructure to provide secured data access to authorized stakeholders (Goodman and Rowland, 2021). Illicit access to signature neural activities mapped to passwords or visual and auditory stimulus and their manipulation can damage an individual's social presence (Armengol-Urpi et al., 2023; Ienca and Haselager, 2016). Wireless BCI systems may be hackable, so adequate cybersecurity measures are essential (Bernal et al., 2023; Ajrawi et al., 2021; Ienca and Haselager, 2016). This becomes concerning if a BCI system has the potential to alter functional neural circuits, posing risks to core aspects of a user's identity such as personality, memory, and emotional regulation (Steinert and Friedrich, 2020; Ienca and Haselager, 2016). Altering human cognitive capacity is challenging because it is unclear when mental changes are reversible (Dunlap et al., 2020; Schmitz-Luhn et al., 2012). Inappropriate use of neural data can distort intended BCI outcomes, potentially leading to unethical or unsafe control of ARTs. Integrated cryptographic encryption and access control into BCI may offer a secure option, fulfilling BCI cybersecurity requirements. Figure 5 illustrates the translational components of BCIs, from technology standardization to ethics policies, algorithm development and essential cybersecurity measures that promote BCIs as promising ARTs for people with disabilities, contributing to their widespread dissemination. The translational elements of BCI can be described from the perspectives of technology, ethics, and cyberspaces. The first includes advanced neural sensors, generalized digital signal processing and pattern recognition algorithms, and individualized ARTs. The elements of ethical space include surgical risk factors associated with electrode implantation, technology standardization, and lawful utilization. Finally, key cybersecurity measures are data confidentiality, availability, and integrity.

## 5 Conclusion

- Recent advancements in neuroimaging and artificial intelligence are rapidly transforming BCI technologies, expanding their potential as ARTs for people with disabilities. BCIs offer novel pathways for communication and control, particularly when conventional methods are ineffective or unavailable.
- To ensure broader accessibility and adoption, the development of generalizable digital signal processing and pattern recognition algorithms remains a critical priority. At the same time, the success of BCI-based ARTs increasingly depends on personalized, user-centered design approaches that reflect the diverse and evolving needs of individuals with disabilities.
- Given the sensitive nature of neural data and its potential to reveal deeply personal information, the ethical, legal, and social implications of BCI use must be addressed proactively. Establishing robust regulatory frameworks and inclusive guidelines–developed in collaboration with all stakeholders–is essential to ensure the responsible and equitable deployment of BCI technologies.
- Ultimately, the future of BCI-based ARTs lies in balancing technological innovation with ethical foresight, personalization with scalability, and accessibility with security. This integrated approach will be key to realizing the full potential of BCIs in enhancing autonomy, communication, and quality of life for people with disabilities.

In summary, researchers and developers must adopt an inclusive and personalized design strategy for BCI-based assistive and rehabilitative applications, engaging all stakeholders from the early stages of the development process. Such a user-centric methodology could maximize the benefit to people with disabilities while maintaining ethical and moral standards for sustainable BCI use for people with disabilities who need this technology most. Future research should consider the opinions of all stakeholders, which will help justify and guide technological advancements in individualized BCI-based ARTs.
